# 2583. Antimicrobial Resistance Profile of *Mycobacterium simiae* and Treatment Trends from a Tertiary Care Center in Lebanon

**DOI:** 10.1093/ofid/ofad500.2198

**Published:** 2023-11-27

**Authors:** Johnny Zakhour, Elio Bitar, Mariam Chalhoub, Saliba Wehbe, Souad Bou Harb, Souha S Kanj

**Affiliations:** American University of Beirut Medical Center, Baabda, Mont-Liban, Lebanon; American University of Beirut Medical Center, Baabda, Mont-Liban, Lebanon; American University of Beirut Medical Center, Baabda, Mont-Liban, Lebanon; American University of Beirut Medical Center, Baabda, Mont-Liban, Lebanon; American University of Beirut Medical Center, Baabda, Mont-Liban, Lebanon; American University of Beirut Medical Center, Baabda, Mont-Liban, Lebanon

## Abstract

**Background:**

*Mycobacterium simiae* is a rare non-tuberculous mycobacterium (NTM) that is most identified in some Middle Eastern countries. It is unclear why this NTM is geographically restricted. The pathogen can either be a mere colonizer or cause significant morbidity. Patients with *M. simiae* infection require prolonged treatment with a combination of 3 antimicrobials. Drug resistance among *M. simiae* isolates can complicate the course of treatment. However, the correlation between *in vitro* resistance and clinical response is unknown. We aimed to report the susceptibility profile of *M. simiae* isolates from our institution and the prescribed treatments.

**Methods:**

We conducted a retrospective study of *M. simiae* isolates from the American University of Beirut Medical Center (AUBMC) over 19 years. All isolates were cultured at AUBMC using the Lowenstein-Jensen media and the BacT/ALERT system then referred for speciation and susceptibility testing at Mayo Clinic, MN, USA.

**Results:**

We included 72 isolates that underwent susceptibility testing from 59 patients. Most isolates were obtained from sputum samples (61.1%) and the rest from bronchoalveolar lavages (BAL). The highest rates of susceptibility were to clarithromycin (84.7%) and amikacin (73.6%). All isolates were resistant to ciprofloxacin, streptomycin, doxycycline, and minocycline (Graph 1). Median minimal inhibitory concentrations (MIC) for clarithromycin and amikacin for susceptible isolates were 8 and 16 mcg/mL respectively. Clofazimine susceptibility was performed on only 16 isolates, 10 of which had an MIC ≤ 0.06 mcg/mL and the highest was 0.25 mcg/mL. Empiric antimicrobial therapy was given to 30% of patients but was inappropriate in 80% of cases. Targeted therapy was given to 40.9% of patients. Most received triple therapy with clarithromycin (95.8%) and clofazimine (41.7%) being the most common agents. The median duration of treatment was 12 months with the longest duration being 11 years. Microbiologic cure was achieved in 73.9% of the 23 patients who underwent repeated testing.

Susceptibility of Mycobacterium simiae isolates according to the CLSI breakpoints.

**Figure:**
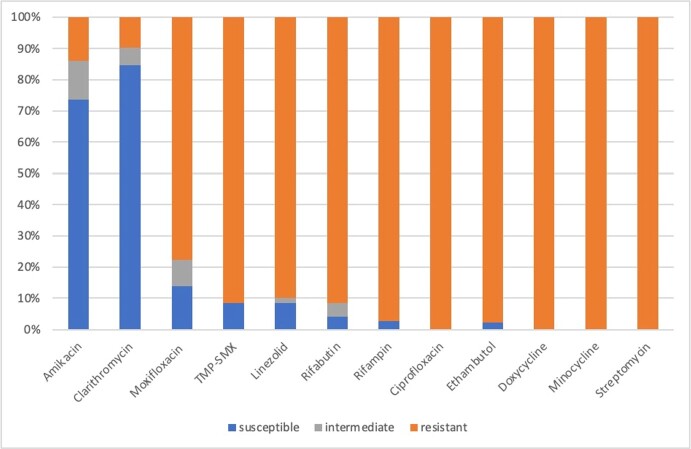

TMP-SMX, trimethoprim-sulfamethoxazole. *Clofazimine was not included in this graph as there is no consensus on breakpoints for susceptibility for NTM. All our isolates had MICs ≤ 0.25 mcg/mL which is lower than breakpoints used in other studies.

**Conclusion:**

*M. simiae* exhibits resistance to most available antimycobacterial agents. Further research is needed to understand resistance mechanisms of this organism and correlate in vitro and in vivo findings.

**Disclosures:**

**Souha S Kanj, MD**, Gilead: Advisor/Consultant|Menarini: Advisor/Consultant|MSD: Advisor/Consultant|Pfizer: Advisor/Consultant

